# Inulin with different degrees of polymerization protects against diet-induced endotoxemia and inflammation in association with gut microbiota regulation in mice

**DOI:** 10.1038/s41598-020-58048-w

**Published:** 2020-01-22

**Authors:** Li-Li Li, Yu-Ting Wang, Li-Meng Zhu, Zheng-Yi Liu, Chang-Qing Ye, Song Qin

**Affiliations:** 1 Yantai Institute of Coastal Zone Research, Chinese Academy of Sciences, Yantai, 264003 China; 2 School of Public Health, Nantong University, Nantong, 226019 China; 3 Center for Ocean Mega-Science, Chinese Academy of Sciences, Qingdao, 266071 China

**Keywords:** Food microbiology, Microbiome

## Abstract

Societal lifestyle changes, especially increased consumption of a high-fat diet lacking dietary fibers, lead to gut microbiota dysbiosis and enhance the incidence of adiposity and chronic inflammatory disease. We aimed to investigate the metabolic effects of inulin with different degrees of polymerization on high-fat diet-fed C57BL/6 J mice and to evaluate whether different health outcomes are related to regulation of the gut microbiota. Short-chain and long-chain inulins exert beneficial effects through alleviating endotoxemia and inflammation. Antiinflammation was associated with a proportional increase in short-chain fatty acid-producing bacteria and an increase in the concentration of short-chain fatty acids. Inulin might decrease endotoxemia by increasing the proportion of *Bifidobacterium* and *Lactobacillus*, and their inhibition of endotoxin secretion may also contribute to antiinflammation. Interestingly, the beneficial health effects of long-chain inulin were more pronounced than those of short-chain inulin. Long-chain inulin was more dependent than short-chain inulin on species capable of processing complex polysaccharides, such as *Bacteroides*. A good understanding of inulin-gut microbiota-host interactions helps to provide a dietary strategy that could target and prevent high-fat diet-induced endotoxemia and inflammation through a prebiotic effect.

## Introduction

The gut microbiota plays an important role in maintaining host health via several pathways, such as enteroendocrine signaling and the gut barrier. The gut microbiota community can be modulated by prebiotics, such as inulins, which allows prebiotics to resist digestion in the upper intestinal tract; thus, most of them are fermented in the colon. A prebiotic is now defined as a substrate that is selectively utilized by host microorganisms and exerts beneficial effects on the host^1^. Inulins, linear carbohydrate polymers that consist of β-(2, 1) fructosyl-fructose linkages, are widely added to dairy products, such as milk drinks and desserts. Because of their selective fermentation feature in the colon, inulins exhibit different characteristics from most dietary fibers^2^; thus, they show beneficial effects through several gastrointestinal functions, including increasing mineral absorption^3^ and systemic immunity^4^, as well as decreasing the risks of intestinal infections^5^, liver diseases^6^, colon cancer^7^, type II diabetes^8^ and obesity^9^.

The high prevalence of obesity and its associated metabolic disorders is currently a major threat to public health in both developing and industrialized countries. Societal lifestyle and dietary changes, especially increased consumption of processed foods and a high-fat diet lacking dietary fibers, are thought to affect the microbiota and enhance the incidence of adiposity and chronic inflammatory disease. In recent decades, the relationship between the gut microbiota and obesity has increasingly aroused general concerns. Analysis of the microbiota from a pair of twins indicated the association between the gut microbiota and obesity-related pathologies^10^. We hypothesize that inulin is beneficial to mice fed a high-fat diet, especially via its prebiotic properties. The prebiotic effect of inulin depends mainly on its degrees of polymerization, which determine its degradation site, hydrolysis rate and fermentation products^11,12^. However, there is little information available on the impact of microbial changes and microbial-dependent metabolites and components driven by inulin on physiological indexes disturbed by a high-fat diet. The objective of this study was to evaluate how inulin with different degrees of polymerization modulated gut microbial ecology and host physiology, including mainly biochemical indicators, glucose metabolism and immunity, as well as to assess whether these microbial changes affect the host phenotype in high-fat diet-fed mice.

## Results

### Food intake and body and tissue weight

**Figure 1:**
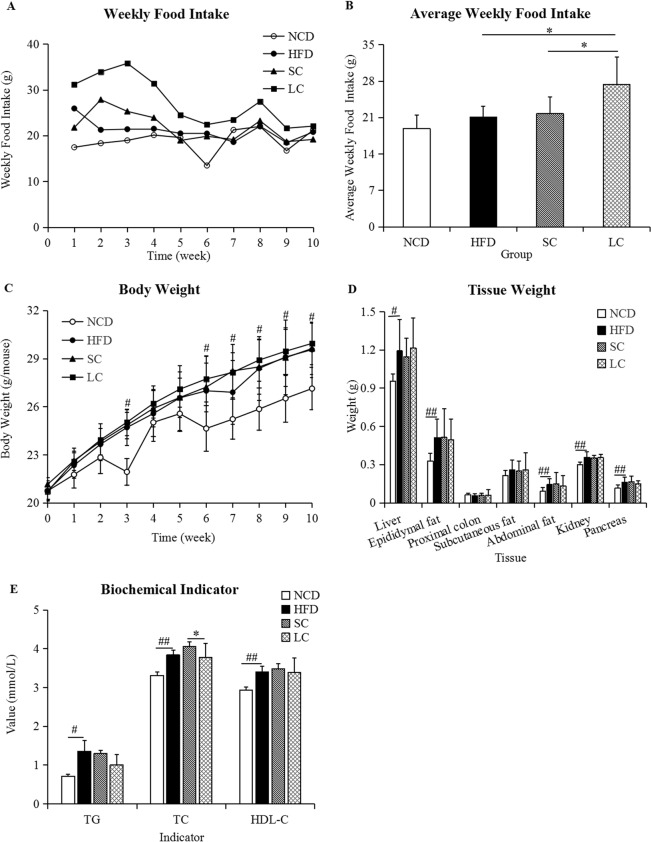
(**A**) Weekly food intake, (**B**) average weekly food intake, (**C**) body weight, (**D**) tissue weight and (**E**) biochemical indicators. Data are represented as the mean ± SD (n = 10). ^#^*p* < 0.05, ^##^*p* < 0.01, comparisons between the HFD and NCD groups; *p < 0.05, **p < 0.01, comparisons among the HFD, SC and LC groups. TG, triacylglycerol; TC, total cholesterol; HDL-C, high-density lipoprotein cholesterol. NCD, normal chow diet; HFD, high-fat diet; SC, high-fat diet plus short-chain inulin; LC, high-fat diet plus long-chain inulin.

Food intake in the LC (high-fat diet plus long-chain inulin) group was always higher than that of the other three groups (Fig. 1A). The average food intakes in the NCD (normal chow diet), HFD (high-fat diet), SC (high-fat diet plus short-chain inulin) and LC groups were 18.89, 21.07, 21.76 and 27.36 g/week, respectively. The average weekly food intake in the LC group was significantly higher than that in the HFD and SC groups (*p* < 0.05), whereas no significant difference was observed between the HFD and NCD groups (Fig. 1B, *p* > 0.05). The body, liver, epididymal fat, abdominal fat, kidney and pancreas weights at the tenth week in the HFD group were significantly higher than those in the NCD group (*p* < 0.05), whereas there were no significant differences among the HFD, SC and LC groups (Fig. 1C,D, *p* > 0.05).

### Biochemical indicators and glycemic metabolism

**Figure 2:**
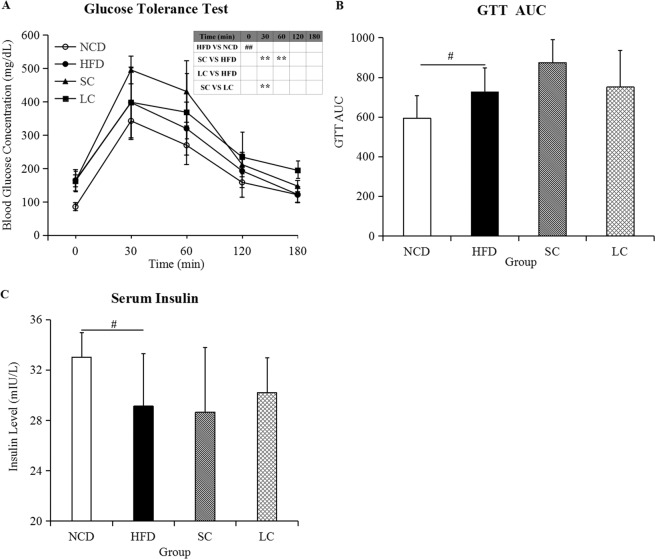
(**A**) Glucose tolerance test, (**B**) glucose tolerance test area under the glucose curve (GTT AUC) and (**C**) the serum insulin level. Data are represented as the mean ± SD (n = 10). ^#^*p* < 0.05, ^##^*p* < 0.01, comparisons between the HFD and NCD groups; *p < 0.05, **p < 0.01, comparisons among the HFD, SC and LC groups. NCD, normal chow diet; HFD, high-fat diet; SC, high-fat diet plus short-chain inulin; LC, high-fat diet plus long-chain inulin.

Biochemical analysis demonstrated that a high-fat diet resulted in a significant increase in serum triacylglycerol (TG), total cholesterol (TC) and high-density lipoprotein cholesterol (HDL-C) compared to those in the NCD group (*p* < 0.05), whereas we observed no significant differences in TG and HDL-C levels among the HFD, SC and LC groups (Fig. 1E, *p* > 0.05). Moreover, lower TC levels were observed in the LC group than in the SC group (Fig. 1E, *p* < 0.05). Higher blood glucose levels were observed in the SC group than in the HFD group at 30 and 60 minutes after glucose loading, and blood glucose concentrations in the SC group were higher than those in the LC group at 30 minutes (Fig. 2A, *p* < 0.01). Furthermore, fasting glucose concentrations and glucose tolerance test area under the glucose curve (GTT AUC) in the HFD group were significantly higher than those in the NCD group (Fig. 2A,B, *p* < 0.05). No significant differences in fasting glucose levels and glucose tolerance test area under the glucose curve (GTT AUC) were observed among the HFD, SC and LC groups (*p* > 0.05). In parallel, serum insulin analysis demonstrated a significant decrease in the HFD group compared with that in the NCD group (*p* < 0.05), and there was no significant difference among the HFD, SC and LC groups (Fig. 2C, *p* > 0.05).

### Immunomodulation

**Figure 3:**
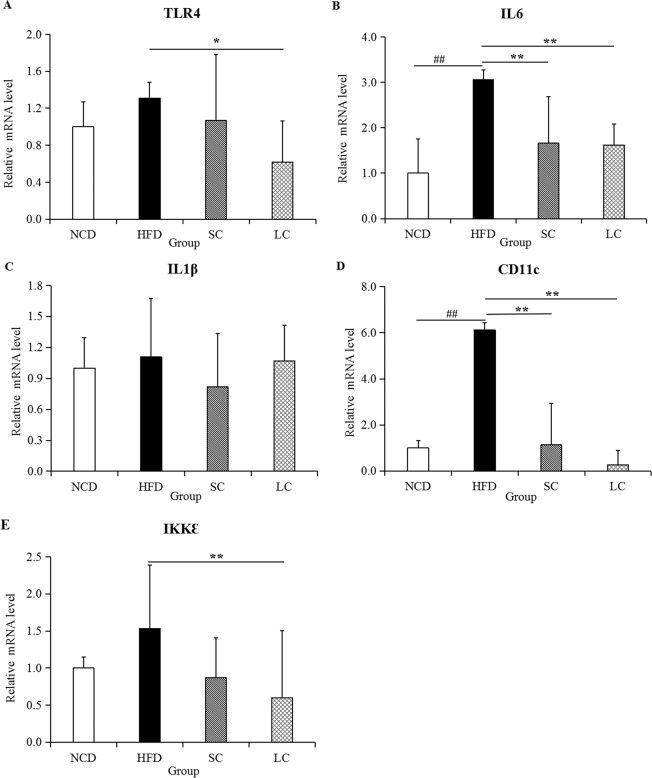
Expression of inflammatory cytokines, including (**A**) TLR4, (**B**) IL6, (**C**) IL1β, (**D**) CD11 and (**E**) IKKε, in epididymal adipose tissue. Data are represented as the mean ± SD (n = 7). ^#^*p* < 0.05, ^##^*p* < 0.01, comparisons between the HFD and NCD groups; *p < 0.05, **p < 0.01, comparisons among the HFD, SC and LC groups. NCD, normal chow diet; HFD, high-fat diet; SC, high-fat diet plus short-chain inulin; LC, high-fat diet plus long-chain inulin.

Transmembrane protein Toll-like receptor 4 (TLR4), interleukin 6 (IL6), interleukin 1β (IL1β), a dendritic cell marker (CD11c) and Ikk kinase ε (IKKε) are highly important proinflammatory indicators, as their abnormal activity is a major feature of inflammation. The mRNA expression of proinflammatory cytokines in epididymal fat tissue was evaluated. Compared to those in the NCD group, TLR4, IL6, IL1β, CD11c and IKKε levels in the HFD group rose 31.49%, 206.19%, 10.79%, 512.62% and 53.10%, respectively (Fig. 3). The five proinflammatory cytokines evaluated in this study were all inhibited to different extents by SC and LC inulin treatment.

### Short-chain fatty acids (SCFAs) and endotoxin

**Figure 4:**
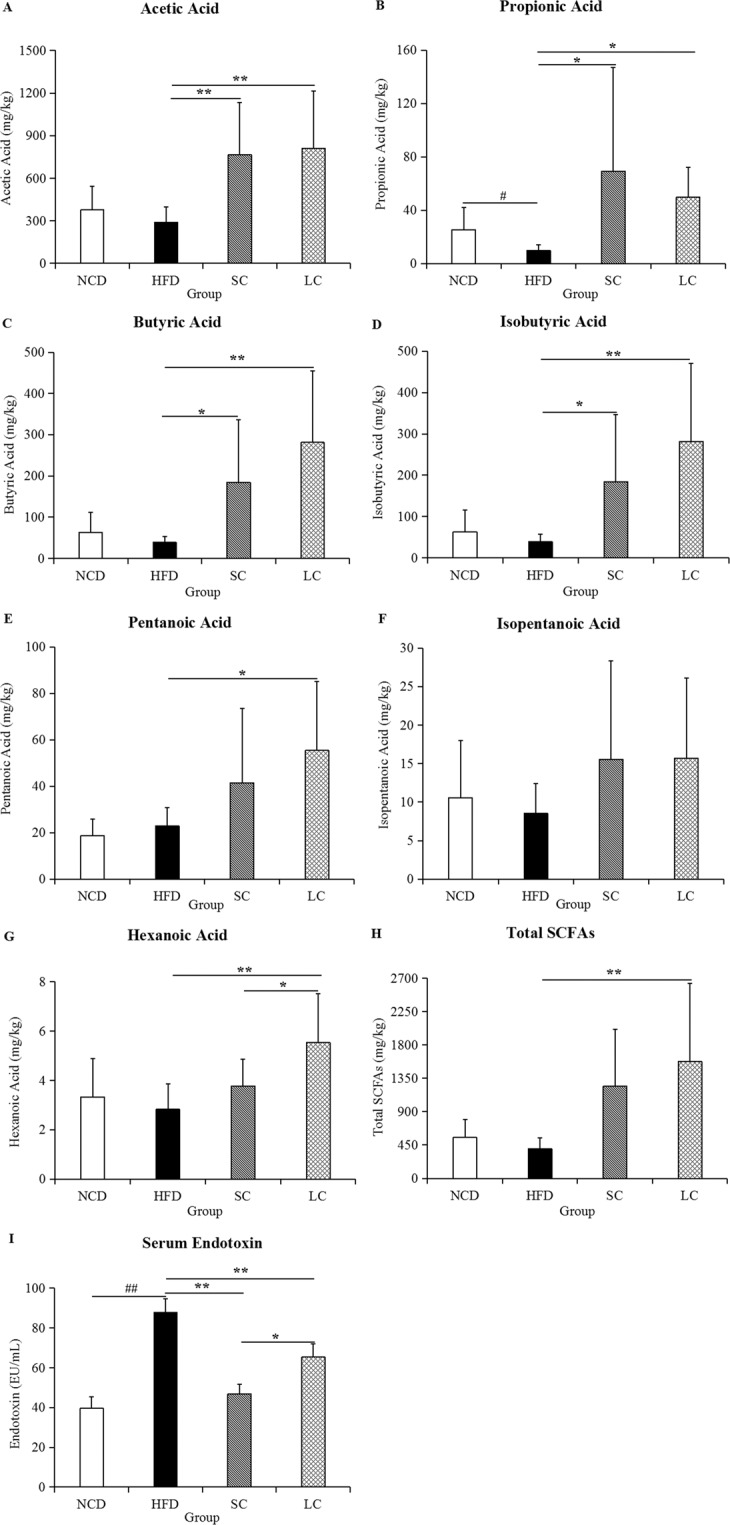
The levels of SCFAs in feces and endotoxin in serum. (**A**) Acetic acid, (**B**) propionic acid, (**C**) butyric acid, (**D**) isobutyric acid, (**E**) pentanoic acid, (**F**) isopentanoic acid, (**G**) hexanoic acid, (**H**) total SCFAs, and (**I**) endotoxin. Data are represented as the mean ± SD (n = 10). ^#^*p* < 0.05, ^##^*p* < 0.01, comparisons between the HFD and NCD groups; *p < 0.05, ** *p* < 0.01, comparisons among the HFD, SC and LC groups. NCD, normal chow diet; HFD, high-fat diet; SC, high-fat diet plus short-chain inulin; LC, high-fat diet plus long-chain inulin.

Compared to the NCD group, the high-fat diet group exhibited reduced concentrations of acetic acid, propionic acid, butyric acid, isobutyric acid, isopentanoic acid, hexanoic acid and total SCFAs by 23.41%, 61.41%, 45.80%, 38.47%, 19.43%, 15.20% and 27.40%, respectively (Fig. 4A–H). However, SC inulin intervention significantly increased the concentrations of acetic acid (*p* < 0.01), propionic acid (*p* < 0.05), butyric acid (*p* < 0.05) and isobutyric acid (*p* < 0.05). The levels of acetic acid, propionic acid, butyric acid, Isobutyric acid, pentanoic acid, hexanoic acid and total SCFAs were 1.81-fold, 4.09-fold, 7.32-fold, 6.27-fold, 1.42-fold, 0.96-fold and 2.94-fold higher, respectively, in the LC group than in the HFD group. Furthermore, compared to those in the NCD group, the levels of endotoxin in the HFD group were increased by 122.00% (Fig. 4I, *p* < 0.01). SC and LC inulin intervention significantly reduced endotoxin levels by 46.77% and 25.65% compared to those in the HFD group, respectively (*p* < 0.01).

### Microbial community analysis

**Figure 5:**
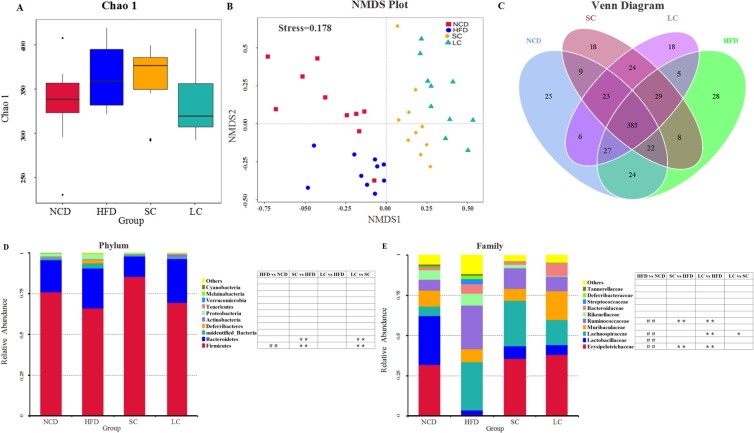
Microbial community analysis. (**A**) Chao 1, (**B**) nonmetric multidimensional scaling (NMDS), (**C**) Venn diagram, (**D**) relative abundance of the top ten phyla, (**E**) relative abundance of the top ten families. n = 10 per group. ^#^*p* < 0.05, ^##^*p* < 0.01, comparisons between the HFD and NCD groups; *p < 0.05, **p < 0.01, comparisons among the HFD, SC and LC groups. NCD, normal chow diet; HFD, high-fat diet; SC, high-fat diet plus short-chain inulin; LC, high-fat diet plus long-chain inulin.

Chao 1, one of the most widely used alpha-diversity indexes in ecology, was used to estimate the total number of species. Our results showed that species richness in the HFD group was higher than that in the NCD group (Fig. 5A). Compared with the HFD, SC inulin increased species richness, whereas LC inulin decreased species richness (Fig. 5A). The difference in samples was analyzed by a nonmetric multidimensional scaling (NMDS) model, which revealed obvious separation among the NCD, HFD and two inulin groups (Fig. 5B). One-way analysis of similarity (ANOSIM) analysis showed that the separation was significant among the four groups (see Supplementary Fig. S1). Our results indicated that distinct diet treatments (NCD, HFD, SC and LC) produced distinct gut microbial communities. Furthermore, a Venn graph exhibited the shared and specific Operational taxonomic units (OTUs) among the four groups (Fig. 5C).

Taxonomic profiling suggested that the gut microbiota structure of mice was dominated mainly by the Firmicutes and Bacteroidetes phyla, which comprised more than 90% of the total phyla in the four groups (Fig. 5D). The ratios of Firmicutes to Bacteroidetes were 3.86, 2.72, 6.87 and 2.59 in the NCD, HFD, SC and LC groups, respectively. Furthermore, a high-fat diet significantly decreased the abundance of the Firmicutes phylum (*p* < 0.01), and this shift was restored by the SC and LC inulin treatments. The dominant families were Erysipelotrichaceae, Lactobacillaceae, Lachnospiraceae, Muribaculaceae, Ruminococcaceae and Rikenellaceae, accounting for more than 75% of the total families (Fig. 5E). The abundances of the Lachnospiraceae and Ruminococcaceae families were significantly stimulated by a high-fat diet (*p* < 0.01), and the enhancement was reduced by the SC and LC inulin interventions. Additionally, the abundances of the Erysipelotrichaceae and Lactobacillaceae families were decreased by a high-fat diet, and this reduction was increased by the SC and LC inulin supplementations. Notably, compared with SC inulin, LC inulin significantly suppressed the growth of the Lachnospiraceae family.

### Insights into the altered gut microbiota

**Figure 6:**
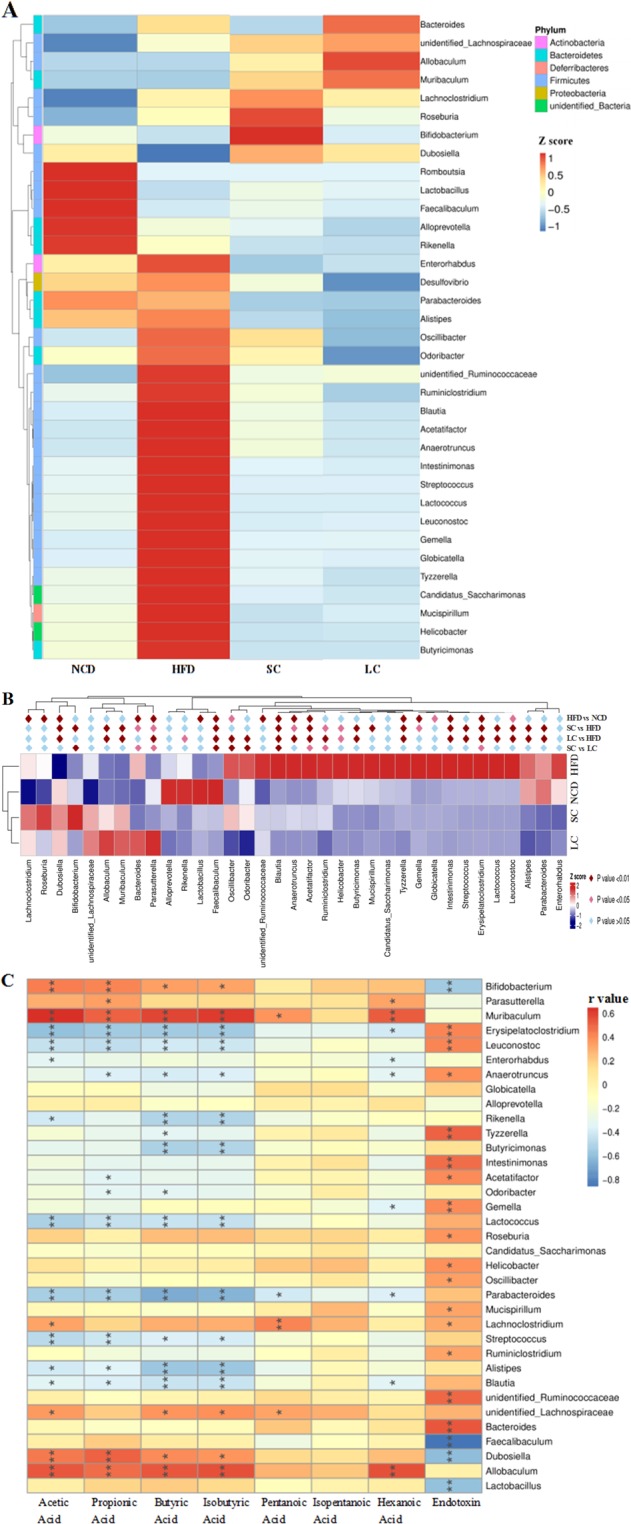
Statistical and correlation analysis of the gut microbiota. (**A**) Different gut microbial phyla among the four groups, (**B**) statistical analysis of all different genera in the four groups and (**C**) Spearman correlations between the levels of metabolites/components and the abundances of gut microbial genera. n = 10 per group. Cells marked with asterisks depict significance, *p < 0.05, **p < 0.01. NCD, normal chow diet; HFD, high-fat diet; SC, high-fat diet plus short-chain inulin; LC, high-fat diet plus long-chain inulin.

A closer look at the microbiota community revealed the top thirty-five OTUs of differentially abundant genera in the four groups, and species belonging to the Firmicutes phylum were the main responders to the high-fat diet and inulin intervention (Fig. 6A). Additionally, abundances of the well-known probiotics *Bifidobacterium* and *Lactobacillus* were decreased by a high-fat diet, and inulin intervention restored this reduction. Further statistical analysis demonstrated that the HFD group had significantly higher abundances of the *Lachnoclostridium*, *Roseburia*, *Bacteroides*, *Oscillibacter*, *unidentified_Ruminococcaceae*, *Blautia*, *Anaerotruncus*, *Acetatifactor*, *Tyzzerella*, *Gemella*, *Globicatella*, *Intestinimonas*, *Erysipelatoclostridium*, and *Leuconostoc* genera and lower abundances of the *Dubosiella*, *Parasutterella*, *Lactobacillus* and *Faecalibaculum* genera than the NCD group (Fig. 6B, *p* < 0.05). Compared to the HFD, SC inulin intervention significantly suppressed the proportions of *Bacteroides*, *Blautia*, *Anaerotruncus*, *Acetatifactor*, *Ruminiclostridium*, *Helicobacter*, *Butyricimonas*, *Mucispirillum*, *Tyzzerella*, *Gemella*, *Intestinimonas*, *Streptococcus*, *Erysipelatoclostridium*, *Lactococcus*, *Leuconostoc*, *Alistipes* and *Parabacteroides* (*p* < 0.05), whereas it significantly increased the proportions of *Dubosiella*, *Bifidobacterium*, *Allobaculum*, *Muribaculum*, *Parasutterella* and *Faecalibaculum* (*p* < 0.05). Compared to the HFD, LC inulin intervention significantly decreased the abundances of the *Rikenella*, *Odoribacter*, *Blautia*, *Anaerotruncus*, *Acetatifactor*, *Ruminiclostridium*, *Helicobacter*, *Butyricimonas*, *Tyzzerella*, *Intestinimonas*, *Streptococcus*, *Erysipelatoclostridium*, *Lactococcus*, *Leuconostoc*, *Alistipes* and *Parabacteroides* genera (*p* < 0.05), whereas it significantly increased the abundances of the *Dubosiella*, *Allobaculum*, *Muribaculum*, *Parasutterella*, and *Faecalibaculum* genera (*p* < 0.05). Furthermore, compared to SC inulin intervention, LC inulin intervention resulted in significantly increased proportions of *Bacteroides*, *Parasutterella* and *Erysipelatoclostridium* (*p* < 0.05), whereas compared to LC inulin intervention, SC inulin intervention resulted in significantly increased abundances of *Bifidobacterium*, *Faecalibaculum*, *Oscillibacter*, *Odoribacter*, *Blautia*, *Acetatifactor* and *Ruminiclostridium* (Fig. 6B, *p* < 0.05).

### Correlation analysis of the gut microbiota

Correlation analysis between specific bacterial genera and metabolic indicators showed that *Bifidobacterium*, *Parasutterella*, *Muribaculum*, *Lachnoclostridium*, *unidentified_Lachnospiraceae*, *Dubosiella* and *Allobaculum* had significant positive relations with SCFAs (*p* < 0.05), whereas *Erysipelatoclostridium*, *Leuconostoc*, *Anaerotruncus*, *Rikenella*, *Tyzzerella*, *Butyricimonas*, *Acetatifactor*, *Odoribacter*, *Gemella*, *Lactococcus*, *Parabacteroides*, *Streptococcus*, *Alistipes* and *Blautia* had significant negative relations with SCFAs (Fig. 6C, *p* < 0.05). Moreover, *Bifidobacterium*, *Faecalibaculum*, *Dubosiella*, and *Lactobacillus* presented negative relations with endotoxin (*p* < 0.05), whereas *Erysipelatoclostridium*, *Leuconostoc*, *Anaerotruncus*, *Tyzzerella*, *Intestinimonas*, *Acetatifactor*, *Gemella*, *Roseburia*, *Helicobacter*, *Oscillibacter*, *Mucispirillum*, *Lachnoclostridium*, *Ruminiclostridium*, *unidentified_Ruminococcaceae* and *Bacteroides* presented positive relations with endotoxin (*p* < 0.05).

## Discussion

A high-fat diet significantly increased body weight and tissue weight. Some metabolic metrics, such as TG, TC and serum insulin, were also disturbed by a high-fat diet. Notably, a high-fat diet elicited significantly different gut microbial communities compared with those with a normal chow diet. An increased ratio of Firmicutes to Bacteroidetes (F/B) was observed in the HFD group, which was supported by a study showing that the F/B ratio in overweight human adults was lower than that in lean controls^13^. A high-fat diet significantly increased the abundances of the Lachnospiraceae and Ruminococcaceae families (Fig. 5E), which are associated with obesity^14,15^.

Our work exhibited a significant enrichment of Erysipelotrichaceae after fermentable dietary fiber SC and LC inulin supplementation. Previous studies have shown positive correlations between Erysipelotrichaceae levels and complex carbohydrate consumption^16^. For example, Cox *et al*. reported that dietary fiber hydroxypropyl methylcellulose increased intestinal Erysipelotrichaceae levels^17^. Specifically, our results supported an association between Erysipelotrichaceae abundance and SCFA levels (see Supplementary Fig. S2), which was consistent with a report showing that Erysipelotrichaceae is an SCFA producer and that some species within this family are butyrate-producing bacteria^17^. Furthermore, the importance of Erysipelotrichaceae in inflammatory responses is highlighted by reports that its abundance has been found to be significantly increased in systemic inflammation in chronic HIV infection^18^, inflammatory bowel disease^19^ and colorectal cancer^20^. The main reason for Erysipelotrichaceae affecting immunity might be because specific taxa of Erysipelotrichaceae are highly immunogenic^21,22^.

The gut microbiota communicates with the host through generated small molecular metabolites. SCFAs, a major class of microbial metabolites, serve as signaling molecules that can directly activate and inhibit endogenous signaling pathways or act as energy resources. SC and LC inulins significantly enhanced the concentrations of acetic acid, propionic acid, butyric acid, isobutyric acid and hexanoic acid in our study. Consistent with the enhancement of SCFA levels, a proportional increase in SCFA-producing bacteria, including the *Bifidobacterium*, *Parasutterella* and *Allobaculum* genera, was observed with SC and LC inulin treatments. Spearman correlation analysis also supported these results that *Bifidobacterium*, *Parasutterella* and *Allobaculum* were all positively correlated with SCFA levels.

SCFAs have broad impacts on a variety of aspects of host health outcomes, and SCFAs facilitate host immunity modulation through several pathways. For example, butyrate downregulated proinflammatory mediators by inhibiting histone deacetylases and dendritic cells^23^. Furthermore, SCFAs were found to exhibit proinflammatory effects by upregulating B cell metabolism, which enhances the systemic generation of IgG and IgA to modulate host immune responses^24^. In general, SCFAs can contribute to antiinflammation and help host defense against pathogens because of their capability to infiltrate into the bloodstream, where they can access G protein-coupled receptors in many tissues or suppress histone deacetylase activity in a variety of cells^25,26^.

Endotoxin refers to lipopolysaccharide (LPS), which is a component of the gram-negative bacterial outer membrane and can enter systemic circulation. In this study, the presence of serum endotoxin, which is known as endotoxemia, was significantly enhanced in the HFD group compared to that in the NCD group. Notably, endotoxemia was significantly alleviated by both SC and LC inulin intervention. The gut microbiota contributes to endotoxin regulation. The abundances of the *Bifidobacterium* and *Lactobacillus* genera, which are involved in the reduction in the intestinal endotoxin concentration and improvement in low grade inflammation^27,28^, were decreased by a high-fat diet and increased by inulins in our results. In agreement, *Bifidobacterium* and *Lactobacillus* abundances exhibited negative correlations with endotoxin levels (Fig. 6A,C). This result indicated that inulin decreased endotoxemia by increasing the proportion of *Bifidobacterium* and *Lactobacillus*. The promotion of these beneficial bacteria might shape environmental conditions (e.g., lowering the pH and increasing the levels of SCFAs) and even further inhibit the growth of some harmful bacteria, such as endotoxin-secreting bacteria.

High-fat diet-induced inflammation was clearly linked to endotoxin secretion^29^. Endotoxin can stimulate the TLR4, an endotoxin receptor found on the surface of many immune cells. TLR4 recruits intracellular adapter molecules to amplify the signal and modulates genes that control the inflammatory response. Leaked endotoxin can trigger adipose inflammation and lead to insulin resistance. Moreover, subcutaneous injections of purified LPS into mice stimulate low-grade inflammation in a manner similar to that of high-fat diet-feeding^30^. This result indicated that inulin modulated immunity through its inhibition of endotoxin secretion.

Compared with SC inulin, LC inulin was superior in glucose homeostasis (Fig. 2A) and TC control (Fig. 1E). SC inulin resulted in an increased glucose peak even compared to that of the HFD, which is a detrimental outcome. In general, the beneficial health effects from LC inulin were more pronounced than those from SC inulin. A similar result was found in an *in vitro*-cultured colon microbiota with the Simulator of the Human Intestinal Microbial Ecosystem (SHIME)^31^. Furthermore, LC inulin intervention exceeded 26.80% of the total level of SCFAs compared with that of the SC inulin intervention, which was consistent with the report that higher polymerization indicates higher acidification activity than lower polymerization in ex *vivo* fermentation^32^. Moreover, there was a significant difference in gut microbial communities between the SC and LC groups (Fig. 5B and see Supplementary Fig. S1). The present study highlights the importance of choosing inulins of proper chain length to achieve good health outcomes.

Further insights into the difference in the gut microbiota showed that SC inulin preferentially stimulated the growth of *Bifidobacterium*, *Faecalibaculum*, *Oscillibacter*, *Odoribacter*, *Blautia*, *Acetatifactor* and *Ruminiclostridium*, and LC inulin preferentially supported the growth of *Bacteroides*, *Parasutterella* and *Erysipelatoclostridium*. This result was exemplified in previous studies showing that *Bifidobacterium* specifically utilized SC inulin but not highly polymerized inulin. *Bifidobacterium* has been reported to possess the enzymatic ability to effectively utilize oligosaccharides, which could be induced by the consumption of galactooligosaccharides and oligofructose^33,34^. Notably, *Bacteroides*, an LC inulin responder, has a series of enzymes that degrade complex polysaccharides into oligosaccharides and monosaccharides^35^. Moreover, LC inulin increased the abundance of the Muribaculaceae family 2-fold more than the SC group (Fig. 5E). Muribaculaceae is a newly proposed family encompassing OTUs previously classified as Porphyromonadaceae in some databases^36^. The abundance of Muribaculaceae, for which the name family S24-7 was previously used, was reported to be increased by inulins in our previous study^37^, and this family was versatile with respect to complex carbohydrate degradation^36^. This result indicated that LC inulin was more dependent on bacteria capable of processing complex polysaccharides than SC inulin because any fermentable carbohydrates, especially highly polymerized inulin, must be hydrolyzed to simple sugars before utilization by bacteria. In addition to the degrading ability, the ability to adsorb inulins, as well as to benefit from metabolites via cross-feeding, also determines the capacity per organism to benefit from inulin.

**Figure 7:**
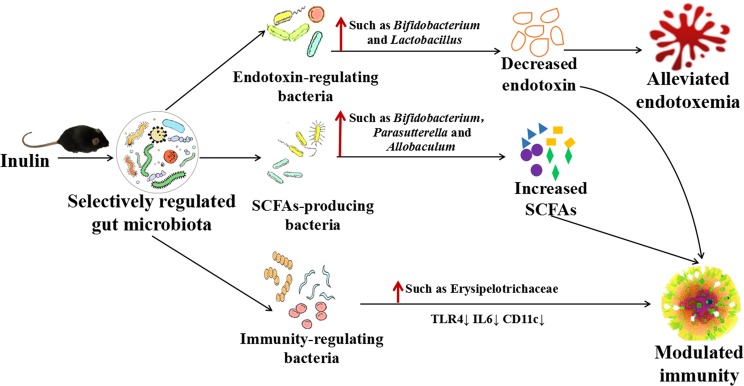
Inulin alleviates endotoxemia and modulates immunity through selectively regulating the gut microbiota in high-fat diet-fed mice.

In conclusion, we found that SC and LC inulin treatments improved host health by alleviating endotoxemia and inflammation in mice fed a high-fat diet (Fig. 7). Notably, the beneficial health effects from LC inulin were more pronounced than those from SC inulin. LC inulin was more dependent on species with efficient hydrolytic capability than SC inulin, such as Bacteroides. Our study further suggests that the abilities of inulin intervention to enhance the relative abundance of SCFA-producing bacteria and increase the levels of SCFAs play a key role in antiinflammation. Inulin might decrease endotoxemia by increasing the proportion of Bifidobacterium and Lactobacillus, and their inhibition of endotoxin secretion also contributed to antiinflammation.

## Materials and Methods

### Materials

Male C57BL/6 J mice were purchased from Pengyue Laboratory Animal Company (Jinan, China). Short-chain inulin (Orafti® P95, the average degree of polymerization is 4-5, purity ≥ 93.2%) and long-chain inulin (Orafti® HP, the average degree of polymerization is 23–25, purity ≥ 99.8%) were procured from Beneo (Tienen, Belgium).

### Animal treatment and sample collection

Mice were housed in a temperature- and humidity-controlled laboratory. Approval of this animal experiment was approved by the Animal Protection Ethics Committee of Binzhou Medical University. The ethical approval number of the animal experiments was F-KY-0022-20181101-01. All animal experiments were performed in accordance with Chinese national regulations on the administration of animal experimentation as well as international guidelines on animal experimentation. After one week of acclimatization, mice were randomly divided into four groups (n = 10): the normal chow diet (NCD, 10% fat calories, Research Diets D12450B; Research Diets, Beijing HFK Bioscience Co., Ltd.) group, high-fat diet (HFD, 60% fat calories, Research Diets D12492; Research Diets, Beijing HFK Bioscience Co., Ltd.) group and high-fat diet plus short-chain inulin (SC, 5 g/100 g diet) or long-chain inulin (LC, 5 g/100 g diet) groups. Food intake and body weight were monitored every week. At 10 weeks, fecal samples were collected in individual sterilized cages and immediately frozen in liquid nitrogen.

### Serum biochemical analysis

Serum samples were separated from blood by centrifugation at 14,000 × g for 15 minutes (4 °C). Biochemical parameters were determined using an automatic analyzer (Roche Diagnostics, Cobas c 311, Switzerland). The parameters analyzed were serum triacylglycerol (TG), total cholesterol (TC) and high-density lipoprotein cholesterol (HDL-C).

### Glucose tolerance test and serum insulin

A glucose tolerance test was carried out at the end of the ninth week of intervention as previously described^38^. Fasting blood glucose (0 minute) was measured after the mice fasted for 15 h. Glucose concentrations were measured at 30 minutes, 60 minutes, 120 minutes and 180 minutes after intraperitoneal injection of glucose (2 g/kg body weight) by using blood glucose meters (Andon Health, Co., Ltd., China). Serum insulin levels were detected using mouse ultrasensitive insulin ELISA kits (Yanbixin, Beijing, China).

### RNA extraction and quantitative real-time RT-PCR

Total RNA from epididymal fat tissue was extracted with TRIzol Reagent (TaKaRa, Japan) and then reverse-transcribed to cDNA with a PrimeScript™ RT Reagent Kit with gDNA Eraser (Perfect Real Time) kit (TaKaRa, Japan). Determinations of mRNA levels were performed with TB Green™ Premix Ex Taq™ II (Tli RNaseH Plus) (TaKaRa, Japan) and a 7500 Fast Real-Time PCR system (ABI, USA). Thermal cycling consisted of an initial cycle of 95 °C for 5 minutes, followed by 40 amplification cycles of 95 °C for 15 s and 60 °C for 1 minute. The target genes were TLR4, IL6, IL1β, CD11c, IKKε and a housekeeping gene (Gapdh), and their primer sequences are listed in Supplementary Table S1.

### Fecal DNA extraction

Total microbial DNA was extracted using a QIAamp DNA Stool Mini Kit (Qiagen, Germany) according to the manufacturer’s instructions. PCR amplification, quantification, and sequencing of 16 S rRNA genes were carried out according to the methods described in our previous study^39^. The library was constructed using Ion Plus Fragment Library Kit 48 reactions (ThermoFisher, USA). After Qubit quantification and testing, the library was sequenced by ThermoFisher’s Ion S5^TM^XL.

### Gut microbiota analysis

Raw data were obtained after data were processed using Cutadapt (V1.9.1, http://cutadapt.readthedocs.io/en/stable/). Then, chimera sequences were removed to obtain clean reads. OTUs were assigned for sequences with ≥ 97% similarity. OTUs were annotated using the SILVA132 database (http://www.arb-silva.de/). The taxonomic information was obtained, and the community composition was counted at seven taxonomic levels: kingdom, phylum, class, order, family, genus and species. Alpha-diversity was analyzed by Chao 1 (http://scikit-bio.org/docs/latest/generated/generated/skbio.diversity.alpha.chao1.html#skbio.diversity.alpha.chao1) with QIIME software (version 1.9.1). Beta-diversity metrics were calculated by the NMDS model based on Bray-Curtis distance. One-way ANOSIM analysis with multiple pairwise post-tests on all groups at the same time was performed to test whether the difference between the extragroups was greater than that between the intragroups and to assess the significance of the difference in separation. The Chao1, Bray-Curtiss indexes, NMDS and ANOSIM were calculated at OTU level. Differentially abundant genera were analyzed by metastats (https://omictools.com/metastats-tool) with a nonparametric test, followed by the Benjamini and Hochberg false discovery rate approach to filter relevant *p*-values.

### Measurement of SCFAs and serum endotoxin

SCFAs, including acetic acid, propionic acid, butyric acid, isobutyric acid, pentanoic acid, isopentanoic acid and hexanoic acid, were extracted by ether (>99%, Sigma Aldrich), with cyclohexanone as an internal standard. The samples were analyzed by gas chromatography-mass spectrometry (GC-MS, Agilent Technologies Inc., Palo Alto, CA, USA). The apparatus parameters were set according to the method described in our previous study^39^. Additionally, serum endotoxin was measured according to the instructions of the Endotoxin ELISA Kit (Yanbixin Company, Beijing, China).

### Statistical analysis

Data were analyzed using SPSS (version 12.0, SPSS Inc., Chicago, IL). Differences between the NCD and HFD groups were analyzed using one-way analysis of variance. The HFD, SC and LC groups were analyzed using one-way analysis of variance and post-hoc testing with the Bonferroni-Holm method. Time-series data from the glucose tolerance test were analyzed using two-way analysis of variance and post hoc testing with the Bonferroni-Holm method. Data are represented as the mean ± SD. *p* values less than 0.05 or 0.01 were considered statistically significant.

## Supplementary information

Supplementary Information.
