# Supplementary material for: Inulin with different degrees of polymerization protects against diet-induced endotoxemia and inflammation in association with gut microbiota regulation in mice
Source: Sci Rep. 2020 Jan 22;10:978. doi: 10.1038/s41598-020-58048-w (PMC6976630; doi:10.1038/s41598-020-58048-w)
Supplement: Supplementary file 1 — Supplementary Information. [file 41598_2020_58048_MOESM1_ESM.pdf]

Supplementary information

Inulin with different degrees of polymerization protects against diet-induced endotoxemia and inflammation in association with gut microbiota regulation in mice

Li-Li Li[1,3], Yu-Ting Wang[1,2,3], Li-Meng Zhu[1,3], Zheng-Yi Liu[1,3], Chang-Qing Ye[2,*] & Song Qin[1,3,*]

[1]Yantai Institute of Coastal Zone Research, Chinese Academy of Sciences, Yantai, 264003, China. [2]School of Public Health, Nantong University, Nantong, 226019, China. [3]Center for Ocean Mega-Science, Chinese Academy of Sciences, Qingdao, 266071, China. Correspondence and requests for materials should be addressed to C.-Q.Y (email: cqye@ntu.edu.cn) or S.Q. (email: sqin@yic.ac.cn).

*Corresponding authors.

Supplementary Table S1. Primers used in this study

Primers	Forward primer	Reverse primer
TLR4	5'-GGGCCTAAACCCAGTCTGTTTG -3'	5'-CTTCTGCCCGGTAAGGTCCA-3'
IL6	5'-CCACTTCACAAGTCGGAGGCTTA -3'	5'-CCAGTTTGGTAGCATCCATCATTTC-3'
IL1β	5'-TCCAGGATGAGGACATGAGCAC -3'	5'-GAACGTCACACACCAGCAGGTTA -3'
CD11c	5'-ACGTCAGTACAAGGAGATGTTGGA -	5'-ATCCTATTGCAGAATGCTTCTTTACC-3'
IKKε	5'-GGAGTGTGTGCAGACGTATCAGG -3'	5'-AATGAGATGCAGGTGGTTCTGG -3'
Gapdh	5'-TGTGTCCGTCGTGGATCTGA -3'	5'-TTGCTGTTGAAGTCGCAGGAG -3'

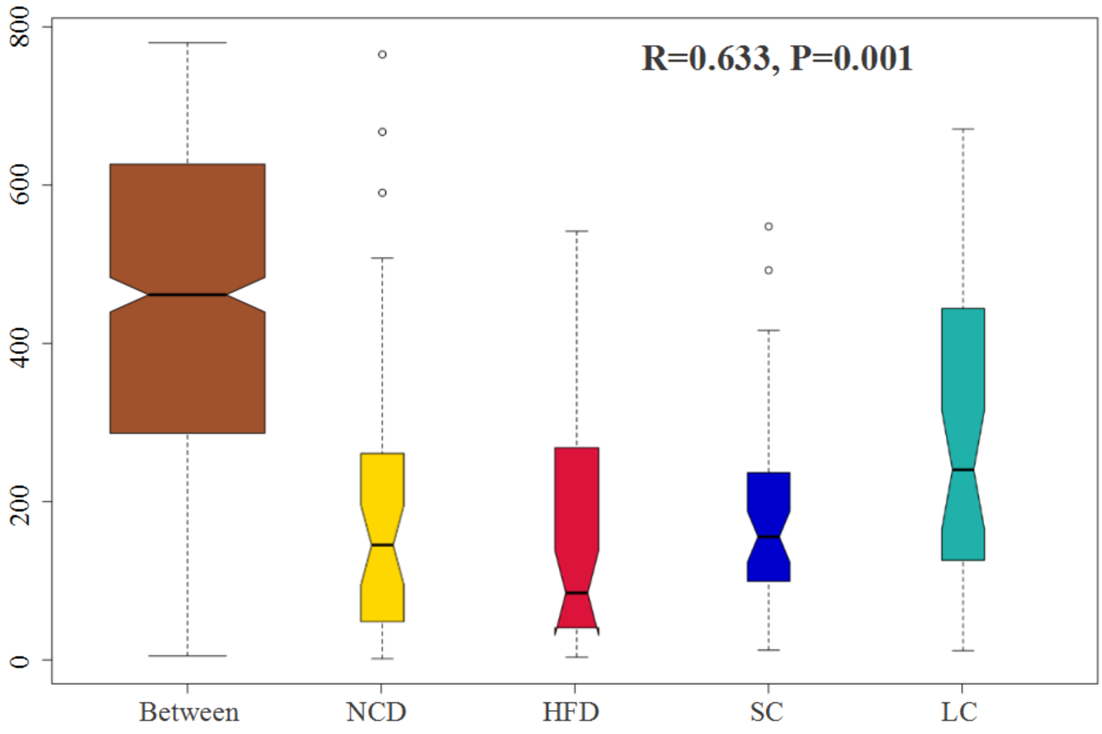

Supplementary Figure S1. One-way ANOSIM analysis of the difference in separation among four groups. NCD, normal chow diet; HFD, high-fat diet; SC, high-fat diet plus short-chain inulin; LC, high-fat diet plus long-chain inulin. R > 0 indicated the difference between the extra-groups was greater than the intra-groups. *p* < 0.05 was considered to be statistically significant.

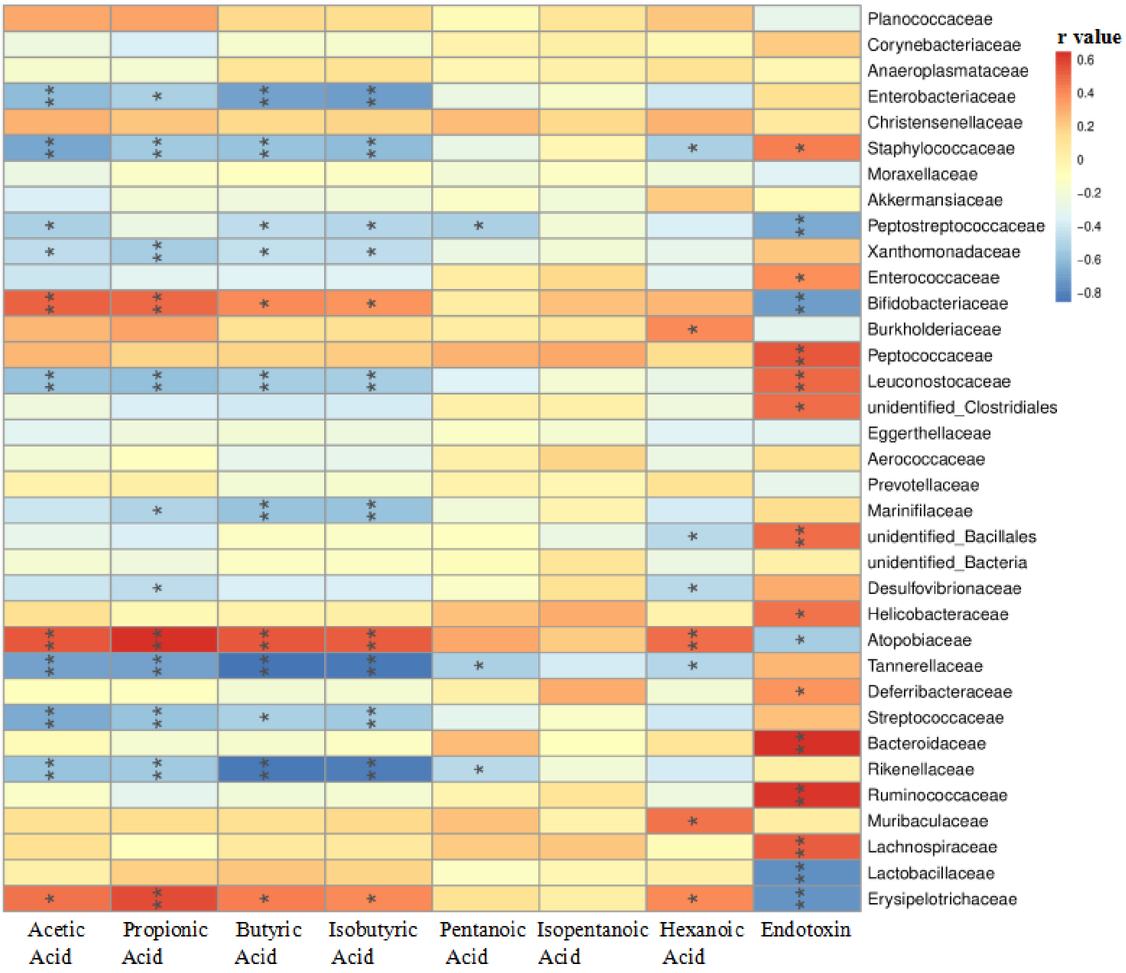

Supplementary Figure S2. Spearman correlations between levels of metabolites/component and abundance of gut microbial families (n = 10). Cells marked with asterisk depict significance, * $p < 0.05$, ** $p < 0.01$.
